# On the Treatment of Placenta Prævia

**Published:** 1846-06

**Authors:** J. J. Tweed

**Affiliations:** M.R.C.S. Eng., London


*On the Treatment of Placenta Prævia.* By J. J. Tweed, Jr., M.R.C.S. Eng., London.—As the question of treatment in cases of placenta prævia is still undecided, and the opinions of many of the most eminent accoucheurs at variance on the point, a case, illustrative of the safety of what may be called the 'new practice,' (although in reality not so), cannot, I think, be otherwise than interesting at the present moment, especially when the importance of the subject is taken into consideration. Statistics have already shown the comparative merits of the two modes of treatment; still, it behooves every one, whatever the practice he adopts, to publish the result, successful or unsuccessful. Where the opportunities for observation occur (fortunately, we may say) so rarely, it is difficult for a man to form an opinion on his own experience alone; on these grounds, I venture to ask a space in your valuable journal for the publication of the following case:—

I was hastily summoned to attend a patient, Mrs. D., aged 22 years, residing in Union-street, in the seventh month of her second pregnancy, (she had a miscarriage, at the fourth month, a year ago,) on Sunday morning November 7th. On my arrival, I learned that labor pains had come on quite suddenly, about nine o'clock, (it was now half past ten), accompanied with profuse discharge which was increased at each pain. She was in a sitting posture, countenance pallid, complained of being 'faintish,' with short pains recurring at intervals of three or four minutes. I immediately had her placed in bed, and on moving her, observed a quantity of coagulated blood on the carpet, over which she had been sitting, and her clothes were also saturated. On examination per vaginam, the uterus was found high up, the os uteri dilated to about the size of a shilling, thick and rigid; passing the finger within the os, the placenta could be distinctly felt, attached immediately over it; each contraction of the uterus gave rise to a gush of blood. I immediately plugged the vagina. At the end of two hours, the plug being removed, the os uteri was found less rigid, thinner, and dilated to the size of a crown piece, each pain forcing the presenting portion of the placenta partly through; as far as the finger could reach, no adhesion of the placenta could be detected. The hemorrhage .was now much diminished; in fact, it had almost entirely ceased. Dr. Murphy, who now kindly saw the case with me, advised again the plugging of the vagina, and if hemorrhage returned to any extent, to introduce the hand, and turn the child immediately. Two hours elapsed, at the expiration of which the plug and placenta were both expelled. No further hemorrhage occurred; the head of the child, with one hand, partly protruded through the os uteri at each pain. I now allowed the labor to proceed naturally for about an hour; the pains then becoming rather inefficient, two doses of the secale cornutum were administered, and the birth of a still-born child took place, exactly an hour after the expulsion of the placenta. The uterus contracted firmly, and the patient is doing well.—*London Lancet.*

